# Effect of esaxerenone on nocturnal blood pressure and natriuretic peptide in different dipping phenotypes

**DOI:** 10.1038/s41440-021-00756-5

**Published:** 2021-10-15

**Authors:** Kazuomi Kario, Sadayoshi Ito, Hiroshi Itoh, Hiromi Rakugi, Yasuyuki Okuda, Satoru Yamakawa

**Affiliations:** 1 Division of Cardiovascular Medicine, Department of Medicine, Jichi Medical University School of Medicine, Shimotsuke, Tochigi Japan; 2 Division of Nephrology, Endocrinology and Vascular Medicine, Department of Medicine, Tohoku University School of Medicine, Sendai, Japan; 3 Katta General Hospital, Shiroishi, Japan; 4 Department of Endocrinology, Metabolism and Nephrology, Keio University School of Medicine, Tokyo, Japan; 5 Department of Geriatric and General Medicine, Osaka University Graduate School of Medicine, Suita, Japan; 6 Daiichi Sankyo Co., Ltd., Tokyo, Japan

**Keywords:** Blood pressure, Esaxerenone, Hypertension, Mineralocorticoid receptor antagonists, NT-proBNP

## Abstract

There are limited data on the nighttime blood pressure (BP)-lowering effect of esaxerenone and its effect on N-terminal pro b-type natriuretic peptide (NT-proBNP), a predictor of cardiovascular risk, according to different dipping patterns of nocturnal BP. This was a post hoc analysis of a multicenter, open-label, long-term phase 3 study of esaxerenone, a new highly selective mineralocorticoid receptor blocker, in patients with essential hypertension. Patients were classified by dipping pattern (extreme dippers, dippers, non-dippers, risers). Mean changes in BP, changes in dipping pattern, mean NT-proBNP levels, and percentage of patients with normal NT-proBNP levels (<55 pg/mL) at baseline and Weeks 12 and 28 were evaluated. Nighttime systolic BP decreased in all dipping pattern groups at Week 28, with the riser group showing the greatest change (−25.5 mmHg). A significant shift in dipping pattern and riser/non-dipper pattern changes to dipper/extreme dipper pattern were found from baseline to Week 28 (*p* < 0.0001). The prevalence of the riser pattern decreased from 14.4% to 9.8%, and that of the non-dipper pattern from 44.7% to 39.2%. The decrease in NT-proBNP from baseline to Week 28 was statistically significant in risers, non-dippers, dippers, and extreme dippers (*p* < 0.001, respectively). At baseline, the proportion of patients with NT-proBNP <55 pg/mL was lowest in risers versus the other dipping pattern types, but after reductions in NT-proBNP in all groups to Week 28, these differences disappeared. Long-term administration of esaxerenone may be a useful treatment option for nocturnal hypertension, especially in patients with a riser pattern.

## Introduction

Blood pressure (BP) normally exhibits diurnal variations. In healthy people, BP tends to decrease (dip) by 10–20% during nighttime (sleep) compared with that during daytime (waking hours) [1]. Based on the night–day BP ratio from 24-h ambulatory blood pressure monitoring (ABPM), variations in BP are classified into four categories: riser, non-dipper, dipper, and extreme dipper [1]. Patients who do not exhibit the usual 10–20% decrease in BP during nighttime are referred to as non-dippers, and those who exhibit an increase in BP during nighttime are referred to as risers. The non-dipper and riser patterns have been associated with a high risk of organ damage to the brain and heart, and cardiovascular death [2–5]. Furthermore, the extreme dipper pattern has been associated with a high risk of stroke [3], impaired cognitive function [6], reduced cerebral blood flow [7], and increased electroencephalographic wave velocity [8].

Nocturnal hypertension (mean nocturnal BP ≥ 120/70 mmHg), which occurs in patients with non-dipper or riser patterns, is an important marker of cardiovascular risk [9–12]. Therefore, understanding the effect of antihypertensive agents on nocturnal hypertension is important to help identify which patients may benefit more from a particular antihypertensive agent.

American, European, and Japanese guidelines recommend brain natriuretic peptide (BNP) and N-terminal pro b-type natriuretic peptide (NT-proBNP) as diagnostic biomarkers for heart failure (HF) [13–15]. In particular, NT-proBNP is considered an indicator of myocardial load as well as a predictor of cardiovascular risk [16, 17]. The results of a previous study suggest that NT-proBNP could be a biomarker for stroke [18], and increased blood levels of NT-proBNP have been reported in patients with symptomatic HF due to left ventricular systolic and diastolic dysfunction [19]. In the J-HOP study of hypertensive patients, patients with higher NT-proBNP levels had a higher incidence of left ventricular hypertrophy or left ventricular space enlargement versus those with lower NT-proBNP levels, suggesting that high NT-proBNP levels may be indicative of organ damage due to elevated nocturnal BP [20]. The J-HOP study also demonstrated a correlation between nocturnal high BP and NT-proBNP [21, 22].

In the PATHWAY-2 study, the mineralocorticoid receptor antagonist spironolactone was found to be an effective treatment option for patients with resistant hypertension [23]. In 2019, esaxerenone, a novel mineralocorticoid receptor blocker, was approved in Japan as a hypertensive drug. Esaxerenone, unlike eplerenone and spironolactone, is more selective due to its non-steroidal structure [24]. In a post hoc analysis of the ESAX-HTN study [25], the nocturnal BP-lowering effect of esaxerenone versus eplerenone was evaluated according to dipping pattern. The results showed nocturnal BP reduction, particularly in patients with non-dipper pattern, after 12 weeks of treatment with esaxerenone or eplerenone; the esaxerenone group showed significant decreases in nocturnal BP compared with eplerenone. However, the nighttime BP-lowering effect of esaxerenone, according to dipping pattern, for longer than 12 weeks has not been sufficiently investigated. In a long-term phase 3 study of esaxerenone in patients with essential hypertension, NT-proBNP levels tended to decrease over the 52-week treatment period; [26] however, to the best of our knowledge, no further studies have verified the effect of esaxerenone on NT-proBNP. Conversely, the effects of finerenone and spironolactone on NT-proBNP have been reported. A study of spironolactone showed no significant difference in NT-proBNP reduction between high-dose and low-dose spironolactone [27], and the ARTS-HF and ARTS-DN studies showed that finerenone decreased BNP and NT-proBNP levels without causing a significant increase in serum potassium levels [28, 29]. This study aimed to examine the effects of esaxerenone on long-term nocturnal BP and NT-proBNP levels according to dipping pattern.

## Methods

### Study design and population

This was a post hoc analysis of a multicenter, open-label, long-term phase 3 study of esaxerenone in patients with essential hypertension (primary study) [26]. The study protocol was approved by the institutional review boards of all participating centers. All procedures were conducted in accordance with the Declaration of Helsinki and Good Clinical Practice, and all patients provided written informed consent.

In the primary study, there was a 4-week observation period, followed by a treatment period from baseline to Week 12 and from Week 12 until Week 28 or Week 52. During the first treatment period, eligible patients received treatment according to the group to which they were allocated: esaxerenone monotherapy, esaxerenone in combination with a renin–angiotensin system (RAS) inhibitor, or esaxerenone in combination with a calcium channel blocker (CCB). Patients received a starting dose of 2.5 mg/day esaxerenone, which was increased to 5 mg/day if required to achieve BP targets as a monotherapy or with a CCB or RAS inhibitor. In the second treatment period (after 12 weeks of treatment), dose adjustments were made, or an additional antihypertensive agent was added as necessary to achieve the target BP. The total treatment period was 28 or 52 weeks. Further details of the study treatment have been published previously [26].

Key inclusion criteria were age ≥20 years, previously untreated essential hypertension or previous treatment with only one RAS inhibitor or CCB at the start of the observation period, sitting systolic BP (SBP) of 140 to <180 mmHg and a sitting diastolic BP (DBP) of 90 to <110 mmHg, 24-h ambulatory BP of ≥130/80 mmHg, and estimated glomerular filtration rate of ≥60 mL/min/1.73 m^2^. Key exclusion criteria were secondary hypertension, orthostatic hypotension, or cardiovascular disease or intervention within the previous 6 months; cerebrovascular disease within the previous year; serum potassium level <3.5 or ≥5.1 mEq/L; or glycated hemoglobin ≥8.4%, type 1 diabetes, and type 2 diabetes with diabetic nephropathy or albuminuria.

For this post hoc analysis, all three treatment groups from the primary study were integrated into a single group and data for up to 28 weeks were used for analysis.

### Outcomes

Sitting SBP (nighttime/daytime) at Weeks 12 and 28 and change from baseline, 24-h average SBP at Weeks 12 and 28 and change from baseline, change in NT-proBNP from baseline to Weeks 12 and 28, proportion of patients with normal NT-proBNP levels (<55 pg/mL) at Weeks 12 and 28 (NT-proBNP level based on a previous study) [30], and change in dipping pattern distribution from baseline to Week 28 were evaluated in this *post hoc* analysis.

### Assessments

Sitting BP was measured at Week 3 and at the end of the observation period (Week 4). During the subsequent treatment period, sitting BP was measured every 2 weeks up to Week 12 and then every 4 weeks up to Week 28 using an automatic BP monitor (HEM-759P Fuzzy device, Omron Healthcare Co., Ltd., Kyoto, Japan). Regarding 24-h BP measurements, these were done at Week 3 during the observation period, and at Weeks 12, 28, and 52 during the treatment period using an ambulatory BP monitor (TM-2433, A & D Co., Ltd., Tokyo, Japan); BP measurements were taken over a minimum of 25 h at 30-minute intervals.

The dipping pattern for each patient was based on their 24-h ABPM recording, and the nighttime SBP dipping (%) was calculated as (1 − average nighttime SBP/average daytime SBP) ×100. The four nighttime BP dipping patterns were defined based on the nighttime SBP dipping (%) as follows: extreme dipper, BP decrease >20%; dipper, ≤20% to >10%; non-dipper, ≤10% to >0%; and riser, ≤0% [31, 32].

To evaluate NT-proBNP levels, blood samples were collected using ethylenediaminetetraacetic acid-2Na vacuum collection tubes. After blood collection, the sample was immediately inverted, mixed, and subjected to centrifugation (4 °C, 3000 rpm, 10 min). The resulting plasma was frozen (≤−20 °C) in a sample container for storage; a minimum volume of 0.8 mL was required for biomarker measurement. Stored samples were processed by an independent laboratory (LSI Medience Corporation, Tokyo, Japan). An electro-chemiluminescence immunoassay was used to measure NT-proBNP levels, with a standard value (minimum amount in plasma that can be measured) of ≤125 pg/mL.

### Statistical analysis

For baseline characteristics, categorical variables are shown as *n* (%) and continuous variables are shown as mean ± SD. Point estimates and corresponding 95% confidence intervals (CI) and *P* values for changes in nocturnal BP and NT-proBNP and for the differences between the dipping patterns were calculated. For NT-proBNP, log-transformed values were used to calculate the geometric mean and geometric mean ratio. *P* values for differences in the proportion of patients with NT-proBNP <55 pg/mL between the four dipping pattern types were tested using analysis of variance. The chi-square test was used to compare differences between the dipping patterns. Bowker’s symmetry test was used for paired categorical variables to compare changes in the distribution of dipping patterns from baseline to Week 28. All tests used a two-sided significance level of 5%, and the multiplicity was not adjusted due to the explanatory nature of this analysis. All statistical analyses were performed using SAS System Release 9.4 (SAS Institute Japan Ltd., Tokyo, Japan).

## Results

### Patients

A total of 368 patients met the inclusion criteria for the primary study and were evaluated. Among these patients, 53 (14.4%), 162 (44.0%), 123 (33.4%), and 29 (7.9%) were categorized as risers, non-dippers, dippers, and extreme dippers, respectively. The mean age of the overall study population was 56.2 years, and the mean 24-h average ambulatory SBP and DBP at baseline were 155.2 and 97.9 mmHg, respectively. Mean age, body mass index, and proportion of female patients were significantly higher in patients with the riser pattern than in those with the dipper pattern (Table 1).

**Table 1:** Baseline demographic and clinical characteristics

	Total (*N* = 368)	Dipping pattern			
		Riser (*N* = 53)	Non-dipper (*N* = 162)	Dipper (*N* = 123)	Extreme dipper (*N* = 29)
Male, *n* (%)	286 (77.7)	32 (60.4)*	130 (80.2)	96 (78.0)	27 (93.1)
Age, years	56.2 ± 9.2	58.6 ± 10.3*	56.6 ± 9.0	54.7 ± 9.0	55.5 ± 8.7
≥65 years, *n* (%)	78 (21.2)	17 (32.1)*	37 (22.8)	20 (16.3)	4 (13.8)
Weight, kg	71.3 ± 12.3	70.4 ± 14.0	71.5 ± 12.8	71.3 ± 11.3	71.8 ± 10.1
Body mass index, kg/m^2^	25.7 ± 3.6	26.8 ± 4.3*	25.6 ± 3.7	25.5 ± 3.1	25.2 ± 3.3
SBP, mmHg	155.2 ± 9.6	157.1 ± 10.4	155.5 ± 9.5	154.1 ± 9.5	155.3 ± 9.2
DBP, mmHg	97.9 ± 5.3	98.3 ± 5.8	98.3 ± 5.2	97.5 ± 5.1	97.6 ± 5.6
24-h average ambulatory SBP, mmHg	159.0 ± 14.1	166.3 ± 16.2	159.2 ± 13.4	157.0 ± 13.6	152.4 ± 11.1
24-h average ambulatory DBP, mmHg	95.5 ± 7.7	97.1 ± 9.0	95.6 ± 7.3	95.4 ± 7.8	92.8 ± 6.6
Hypertension grade, n (%)					
Grade I	176 (47.8)	25 (47.2)	70 (43.2)	65 (52.8)	15 (51.7)
Grade II	192 (52.2)	28 (52.8)	92 (56.8)	58 (47.2)	14 (48.3)
Prior treatment for hypertension, *n* (%)	244 (66.3)	35 (66.0)	110 (67.9)	76 (61.8)	22 (75.9)
Diabetes, *n* (%)	67 (18.2)	9 (17.0)	24 (14.8)	29 (23.6)	4 (13.8)
Serum K^+^, mEq/L	4.17 ± 0.27	4.09 ± 0.26	4.20 ± 0.27	4.17 ± 0.27	4.19 ± 0.27
≥4.5 mEq/L, *n* (%)	58 (15.8)	5 (9.4)	31 (19.1)	17 (13.8)	5 (17.2)
eGFR, mL/min/1.73 m^2^	79.6 ± 12.7	77.2 ± 12.0	79.2 ± 12.8	80.5 ± 12.6	83.2 ± 13.6
HbA1c, %	5.78 ± 0.61	5.71 ± 0.55	5.77 ± 0.55	5.83 ± 0.71	5.68 ± 0.60
NT-proBNP, pg/mL	73.7 ± 61.6	73.7 ± 61.6	58.0 ± 58.5	57.1 ± 64.4	47.1 ± 28.0
<55 pg/mL, *n* (%)	229 (62.2)	24 (45.3)*	103 (63.6)	82 (66.7)	19 (65.5)
Add-on therapy with other antihypertensive agents	123 (33.4)	14 (26.4)	57 (35.2)	41 (33.3)	11 (37.9)
Calcium channel blocker	59 (16.0)	7 (13.2)	29 (17.9)	16 (13.0)	7 (24.1)
RAS inhibitor	64 (17.4)	7 (13.2)	28 (17.3)	25 (20.3)	4 (13.8)
Esaxerenone dose, mg/day					
By week 12	3.51 (0.79)	3.56 (0.82)	3.51 (0.77)	3.46 (0.80)	3.60 (0.78)
By week 28	3.91 (0.94)	3.94 (0.96)	3.92 (0.94)	3.85 (0.95)	3.96 (0.95)

Data are presented as *n* (%) or mean ± standard deviation

*p < 0.05 versus patients with a dipper pattern

*DBP* diastolic blood pressure, *eGFR* estimated glomerular filtration rate, *HbA1c* glycated hemoglobin, *K* potassium, *NT-proBNP* N-terminal pro b-type natriuretic peptide, *RAS* renin–angiotensin system, *SBP* systolic blood pressure

### Outcomes

**Fig. 1:**
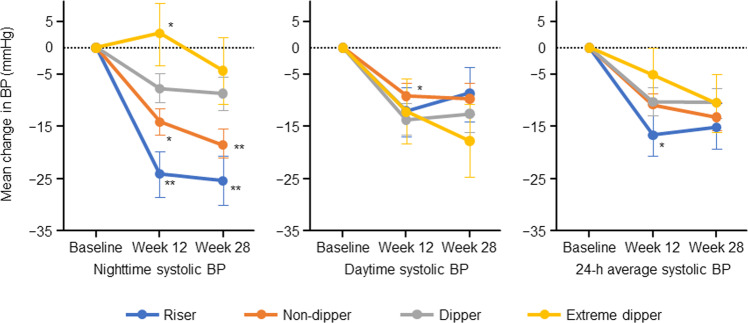
Mean change from baseline in blood pressure. Data are shown as mean ± 95% confidence intervals. The *P* values for the differences between groups (versus dippers): *p < 0.05, **p < 0.001. BP blood pressure, h hour

The mean ± 95% CI changes in nighttime and daytime SBP are shown in Fig. 1. Nighttime SBP decreased from baseline to Week 12 in all dipping pattern groups, except in the extreme dipper type. Nighttime and daytime SBP decreased from baseline to Week 28 in all dipping pattern groups. For nighttime SBP, the decrease at Week 28 was the greatest in the riser type, followed by the non-dipper, dipper, and extreme dipper types. The respective mean changes in nighttime SBP at Weeks 12 and 28 were −24.3 (95% CI −28.7, −19.9) and −25.5 (95% CI −30.3, −20.7) mmHg in the riser, −14.2 (95% CI −16.7, −11.6) and −18.6 (95% CI −21.3, −15.8) mmHg in the non-dipper, −7.7 (95% CI −10.6, −4.8) and −8.8 (95% CI −12.0, −5.7) mmHg in the dipper, and 2.7 (95% CI −3.3, 8.6) and −4.3 (95% CI −10.9, 2.2) mmHg in the extreme dipper types.

In risers, the mean nighttime SBP decreased from 169.5 mmHg at baseline to 145.3 and 143.4 mmHg at Weeks 12 and 28; that in non-dippers decreased from 153.1 mmHg at baseline to 138.8 and 134.5 mmHg; that in dippers decreased from 142.4 mmHg at baseline to 134.6 and 133.4 mmHg; and that in extreme dippers increased from 129.0 mmHg at baseline to 131.8 mmHg at Week 12 and then decreased to 124.7 mmHg at Week 28, respectively. The 24-h average SBP decreased the most in the riser type compared with the other dipping pattern types.

A significant change was observed in the dipping pattern distribution from baseline to Week 28 (*p* < 0.0001) (Table 2). The proportion of patients with the riser type decreased from 14.4% at baseline to 9.8% at Week 28 and that of the non-dipper type decreased from 44.7% at baseline to 32.3% at Week 28.

**Table 2:** Dipping pattern distribution change from baseline to Week 28

		Baseline				
		Riser	Non-dipper	Dipper	Extreme dipper	Total
Week 28	Riser	16 (4.6%)	10 (2.9%)	8 (2.3%)	0 (0%)	34 (9.8%)
	Non-dipper	18 (5.2%)	59 (17.0%)	29 (8.4%)	6 (1.7%)	112 (32.3%)
	Dipper	14 (4.0%)	61 (17.6%)	50 (14.4%)	11 (3.2%)	136 (39.2%)
	Extreme dipper	2 (0.6%)	25 (7.2%)	28 (8.1%)	10 (2.9%)	65 (18.7%)
	Total	50 (14.4%)	155 (44.7%)	115 (33.1%)	27 (7.8%)	347 (100%)

Data are presented as *n* (%)

Bower’s symmetry test: *p* < 0.001

**Fig. 2:**
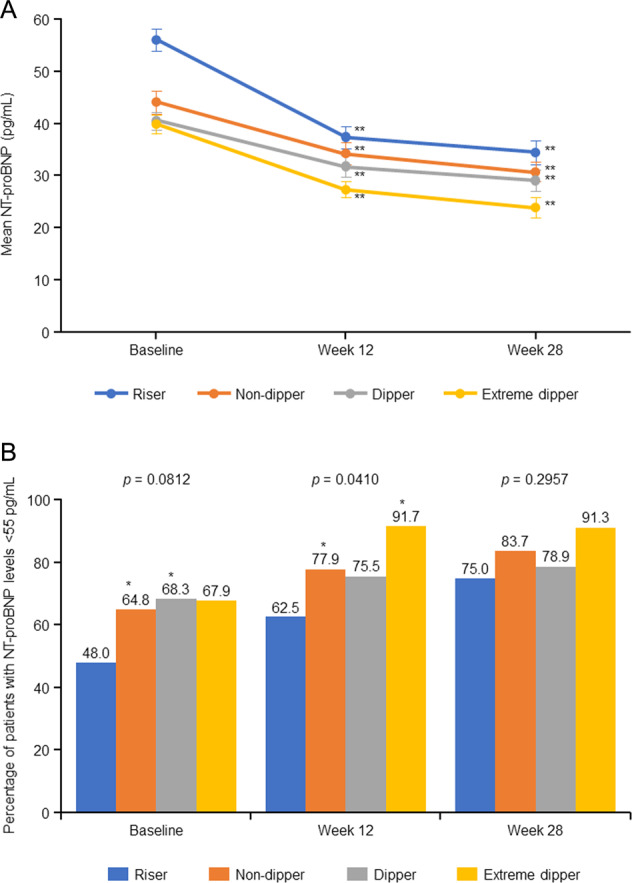
Geometric mean change in NT-proBNP (**A**) and proportion of patients with NT-proBNP level <55 pg/mL (**B**). **A**: Data are shown as point estimates ± 95% confidence intervals. The *p* value versus baseline: **p < 0.001. **B**: The *p* values for the differences between groups (versus risers): *p < 0.05; and the *p* values on the top of the bar graphs were calculated by analysis of variance. NT-proBNP N-terminal pro b-type natriuretic peptide

NT-proBNP levels decreased from baseline to Weeks 12 and 28 in all dipping pattern groups (Fig. 2A). The decrease in NT-proBNP from baseline to Week 28 was statistically significant in the four dipping pattern types: risers (change from baseline −21.9 pg/mL, geometric mean ratio 0.623 [95% CI 0.522, 0.743], *p* < 0.001), extreme dippers (−16.0 pg/mL, 0.588 [95% CI 0.457, 0.757], *p* < 0.001), non-dippers (−13.6 pg/mL, 0.664 [95% CI 0.599, 0.736], *p* < 0.001), and dippers (−10.0 pg/mL, 0.705 [95% CI, 0.626, 0.795], *p* < 0.001). At Week 28, NT-proBNP levels were the highest in the riser and non-dipper types compared with the other dipping pattern types; however, NT-proBNP levels were <55 pg/mL in all groups at Week 28.

The proportion of patients with NT-proBNP <55 pg/mL at baseline and Weeks 12 and 28 are shown in Fig. 2B. At baseline, this proportion was lowest in risers compared with the other dipping pattern types, but these differences disappeared by Week 28 owing to reductions in NT-proBNP being more pronounced in those with the riser pattern.

## Discussion

**Fig. 3:**
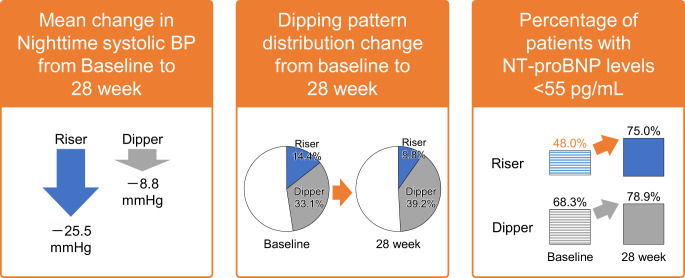
Graphical Abstract: Long-term administration of esaxerenone may be a useful treatment option for nocturnal hypertension, with improving NT-proBNP levels, especially in patients with a riser pattern

In this study, we examined the nighttime BP-lowering effect of esaxerenone at 28 weeks in Japanese patients with hypertension according to their dipping pattern. To the best of our knowledge, this is the first report to examine the effect of esaxerenone on NT-proBNP according to dipping pattern. This post hoc analysis showed that (1) the decrease in nocturnal BP at Week 28 was greatest in the riser type, (2) in all four dipping pattern types, NT-proBNP levels were significantly lower at Week 28 compared with baseline, and (3) a significant change in the dipping pattern distribution from baseline to Week 28 was confirmed. The proportion of patients with riser type and non-dipper type decreased from 14.4% and 44.7% at baseline to 9.8% and 32.3% at Week 28, respectively (Fig. 3).

The decreasing nighttime BP of patients with the riser pattern in this study was similar to that reported in a previous study after 12 weeks of esaxerenone treatment [25]. Furthermore, our findings confirmed that the antihypertensive effect of esaxerenone by dipping pattern is maintained in the long-term up to 28 weeks. Our previous study did not include patients who were receiving treatment with more than one antihypertensive agent [26]. In this study, 123 of 368 patients (33.4%) received add-on therapy with other antihypertensive agents (RAS inhibitor in 64 patients and CCB in 59 patients). Our findings revealed that similar results were observed when esaxerenone was added to these therapies. In addition, a randomized controlled trial comparing eplerenone and placebo as an add-on to other hypertensive treatments in patients with obstructive sleep apnea reported an increase in night BP reduction from 4.6 to 8.9% [33]. Our study showed that at Week 28, the decrease in nocturnal BP was the greatest in risers (−25.5 mmHg) and the lowest in extreme dippers (−4.3 mmHg). This was consistent with the change in distribution from riser/non-dipper to dipper/extreme dipper (Table 2).

The strong mineralocorticoid receptor inhibitory action of esaxerenone [34] could contribute to the decrease in nocturnal BP, leading to a decrease in the cardiovascular risk of esaxerenone-treated patients. This effect on nocturnal BP is of particular relevance in Asian patients, given their higher prevalence of hypertension-related stroke and HF compared with non-Asian patients [35, 36].

The main causes of nocturnal hypertension include advanced structural vascular disease, increased salt sensitivity, and a diet high in salt, particularly in patients with increasing basal nighttime BP [37]. Renal dysfunction, sympathetic hyperactivity, and activation of the renin–angiotensin–aldosterone system all contribute to increased salt sensitivity [37]. Therefore, the mechanism through which esaxerenone decreases nocturnal BP may be explained via its antimineralocorticoid activity.

NT-proBNP levels may indicate subclinical target organ damage to the heart and kidneys in hypertensive patients [38]. A previous study demonstrated that the riser pattern might predict cardiovascular outcomes in patients with HF with preserved ejection fraction [39]. In the present study, NT-proBNP levels at baseline were highest in the riser type compared with the other dipping pattern types, suggesting the possibility of more target organ damage in the riser group at baseline compared with the other dipping pattern groups. In addition, NT-proBNP levels decreased significantly in patients with all four dipping pattern types after 28 weeks of treatment with esaxerenone. This indicates that the myocardial load was reduced due to the decrease in nighttime BP resulting from the mineralocorticoid receptor antagonism. Based on these findings, we consider that NT-proBNP may be a useful indicator of organ damage, especially in the riser dipper type. Furthermore, in patients with high NT-proBNP levels, esaxerenone may be an advantageous treatment option.

As far as we know, there are no previous reports on antihypertensive agents that change the dipping pattern distribution. In the analysis of dipping pattern distribution change from baseline, the proportions of patients with riser or non-dipper types decreased at Week 28. Considering the high risk of organ damage to the brain and heart and cardiovascular mortality associated with non-dipper and riser patterns [2–5], antihypertensive agents with BP-lowering effects in patients with these dipping patterns would be of great value. Our results suggest that esaxerenone may be a suitable treatment option for patients with riser and non-dipper patterns.

Although the mechanism that causes hypertension-mediated organ damage remains unclear, one study suggested that increases in BP may lead to chronic inflammation through local activation of the angiotensin/aldosterone system/mineralocorticoid receptor system [40]. To date, three mechanisms have been proposed in the literature for the association between riser pattern and HF, independent of 24-hour, daytime, and nighttime BP, indicating a “beyond BP” pathophysiological mechanism [41]. The first mechanism involves a vascular component based on the association between the riser pattern and advanced vascular disease (e.g., endothelial dysfunction, increased arterial stiffness). The second mechanism involves circulating volume, based on the association between non-dipping and riser patterns and increased circulating volume, mainly affected by salt sensitivity and salt intake. The third mechanism involves sympathetic nerve activity, given the high muscle sympathetic nerve traffic that characterizes the riser pattern and renal denervation, significantly reducing 24-hour BP, including nighttime BP. All of these factors increase preload/afterload and contribute to the development of HF.

The present study has some limitations. This study was a post hoc analysis with no pre-specified data plan. As there was no comparator agent and only comparisons with the baseline values were determined, the regression to the mean was not considered. This study was conducted in Japanese patients only; thus, generalizability to other ethnic populations may be limited. Finally, the sample size for risers and extreme dippers was very small.

In conclusion, long-term administration of esaxerenone may be a useful treatment option for nocturnal hypertension, especially in patients with a riser pattern.

## Data Availability

De-identified individual participant data and applicable supporting clinical study documents may be available upon request at https://vivli.org/. In cases where clinical study data and supporting documents are provided pursuant to our company policies and procedures, Daiichi Sankyo will continue to protect the privacy of our clinical study participants. Details on data sharing criteria and the procedure for requesting access are available at https://vivli.org/ourmember/daiichi-sankyo/.

## References

[1] Fagard RH (2009). Dipping pattern of nocturnal blood pressure in patients with hypertension. Expert Rev Cardiovasc Ther.

[2] Kario K, Matsuo T, Kobayashi H, Imiya M, Matsuo M, Shimada K (1996). Nocturnal fall of blood pressure and silent cerebrovascular damage in elderly hypertensive patients. Advanced silent cerebrovascular damage in extreme dippers. Hypertension.

[3] Kario K, Pickering TG, Matsuo T, Hoshide S, Schwartz JE, Shimada K (2001). Stroke prognosis and abnormal nocturnal blood pressure falls in older hypertensives. Hypertension.

[4] Kario K, Shimada K (2004). Risers and extreme-dippers of nocturnal blood pressure in hypertension: Antihypertensive strategy for nocturnal blood pressure. Clin Exp Hypertens.

[5] Ohkubo T, Hozawa A, Yamaguchi J, Kikuya M, Ohmori K, Michimata M (2002). Prognostic significance of the nocturnal decline in blood pressure in individuals with and without high 24-h blood pressure: the Ohasama study. J Hypertens.

[6] Guo H, Tabara Y, Igase M, Yamamoto M, Ochi N, Kido T (2010). Abnormal nocturnal blood pressure profile is associated with mild cognitive impairment in the elderly: the J-SHIPP study. Hypertens Res.

[7] Siennicki-Lantz A, Reinprecht F, Axelsson J, Elmståhl S (2007). Cerebral perfusion in the elderly with nocturnal blood pressure fall. Eur J Neurol.

[8] Amah G, Ouardani R, Pasteur-Rousseau A, Voicu S, Safar ME, Kubis N (2017). Extreme-dipper profile, increased aortic stiffness, and impaired subendocardial viability in hypertension. Am J Hypertens.

[9] Drawz PE, Rosenthal N, Babineau DC, Rahman M (2010). Nighttime hospital blood pressure - a predictor of death, ESRD, and decline in glomerular filtration rate. Ren Fail.

[10] Hansen TW, Li Y, Boggia J, Thijs L, Richart T, Staessen JA (2011). Predictive role of the nighttime blood pressure. Hypertension.

[11] Kario K, Williams B (2021). Nocturnal hypertension and heart failure: mechanisms, evidence, and new treatments. Hypertension.

[12] Fujiwara T, Hoshide S, Kanegae H, Kario K (2020). Cardiovascular event risks associated with masked nocturnal hypertension defined by home blood pressure monitoring in the J-HOP nocturnal blood pressure study. Hypertension.

[13] Ponikowski P, Voors AA, Anker SD, Bueno H, Cleland JG, Coats AJ (2016). 2016 ESC guidelines for the diagnosis and treatment of acute and chronic heart failure: The task force for the diagnosis and treatment of acute and chronic heart failure of the European Society of Cardiology (ESC). Developed with the special contribution of the Heart Failure Association (HFA) of the ESC. Eur J Heart Fail.

[14] Yancy CW, Jessup M, Bozkurt B, Butler J, Casey DE, Colvin MM (2017). 2017 ACC/AHA/HFSA focused update of the 2013 ACCF/AHA guideline for the management of heart failure: a report of the American College of Cardiology/American Heart Association task force on clinical practice guidelines and the Heart Failure Society of America. J Am Coll Cardiol.

[15] Tsutsui H, Isobe M, Ito H, Ito H, Okumura K, Ono M (2019). JCS 2017/JHFS 2017 guideline on diagnosis and treatment of acute and chronic heart failure—digest version. Circ J.

[16] Rudolf H, Mügge A, Trampisch HJ, Scharnagl H, März W, Kara K (2020). NT-proBNP for risk prediction of cardiovascular events and all-cause mortality: the getABI-study. Int J Cardiol Heart Vasc.

[17] Yamanouchi S, Kudo D, Endo T, Kitano Y, Shinozawa Y (2010). Blood N-terminal proBNP as a potential indicator of cardiac preload in patients with high volume load. Tohoku J Exp Med.

[18] Doi Y, Ninomiya T, Hata J, Hirakawa Y, Mukai N, Ikeda F (2011). N-terminal pro-brain natriuretic peptide and risk of cardiovascular events in a japanese community: the Hisayama study. Arterioscler Thromb Vasc Biol.

[19] Don-Wauchope AC, Santaguida PL, Oremus M, McKelvie R, Ali U, Brown JA (2014). Incremental predictive value of natriuretic peptides for prognosis in the chronic stable heart failure population: A systematic review. Heart Fail Rev.

[20] Hoshide S, Nagai M, Yano Y, Ishikawa J, Eguchi K, Kario K (2014). Association of high-sensitivity cardiac troponin T and N-terminal pro-brain-type natriuretic peptide with left ventricular structure: J-HOP study. J Clin Hypertens (Greenwich).

[21] Kario K, Hoshide S, Haimoto H, Yamagiwa K, Uchiba K, Nagasaka S (2015). Sleep blood pressure self-measured at home as a novel determinant of organ damage: Japan morning surge home blood pressure (J-HOP) study. J Clin Hypertens (Greenwich).

[22] Hoshide S, Kanegae H, Kario K (2021). Nighttime home blood pressure as a mediator of N-terminal pro-brain natriuretic peptide in cardiovascular events. Hypertens Res.

[23] Williams B, MacDonald TM, Morant S, Webb DJ, Sever P, McInnes G (2015). Spironolactone versus placebo, bisoprolol, and doxazosin to determine the optimal treatment for drug-resistant hypertension (PATHWAY-2): A randomised, double-blind, crossover trial. Lancet.

[24] Daiichi Sankyo Co., Ltd. Esaxerenone (minnebro): Japanese prescribing information; approved January 2019. https://www.Pmda.Go.Jp/drugs/2019/p20190109003/430574000_23100amx00011000_a100_1.Pdf. Accessed 3 June 2021.

[25] Kario K, Ito S, Itoh H, Rakugi H, Okuda Y, Yoshimura M (2021). Effect of the nonsteroidal mineralocorticoid receptor blocker, esaxerenone, on nocturnal hypertension: A post hoc analysis of the ESAX-HTN study. Am J Hypertens.

[26] Rakugi H, Ito S, Itoh H, Okuda Y, Yamakawa S (2019). Long-term phase 3 study of esaxerenone as mono or combination therapy with other antihypertensive drugs in patients with essential hypertension. Hypertens Res.

[27] Butler J, Anstrom KJ, Felker GM, Givertz MM, Kalogeropoulos AP, Konstam MA (2017). Efficacy and safety of spironolactone in acute heart failure: The ATHENA-HF randomized clinical trial. JAMA Cardiol.

[28] Bakris GL, Agarwal R, Chan JC, Cooper ME, Gansevoort RT, Haller H (2015). Effect of finerenone on albuminuria in patients with diabetic nephropathy: a randomized clinical trial. JAMA.

[29] Filippatos G, Anker SD, Böhm M, Gheorghiade M, Køber L, Krum H (2016). A randomized controlled study of finerenone vs. eplerenone in patients with worsening chronic heart failure and diabetes mellitus and/or chronic kidney disease. Eur Heart J.

[30] Hata A, Kiyohara H (2015). Biomarker for stroke: Hisayama Study [in Japanese]. Stroke.

[31] Parati G, Stergiou G, O’Brien E, Asmar R, Beilin L, Bilo G (2014). European Society of Hypertension practice guidelines for ambulatory blood pressure monitoring. J Hypertens.

[32] Umemura S, Arima H, Arima S, Asayama K, Dohi Y, Hirooka Y (2019). The Japanese Society of Hypertension guidelines for the management of hypertension (JSH 2019). Hypertens Res.

[33] Krasińska B, Cofta S, Szczepaniak-Chicheł L, Rzymski P, Trafas T, Paluszkiewicz L (2019). The effects of eplerenone on the circadian blood pressure pattern and left ventricular hypertrophy in patients with obstructive sleep apnea and resistant hypertension-A randomized, controlled trial. J Clin Med.

[34] Arai K, Homma T, Morikawa Y, Ubukata N, Tsuruoka H, Aoki K (2015). Pharmacological profile of CS-3150, a novel, highly potent and selective non-steroidal mineralocorticoid receptor antagonist. Eur J Pharm.

[35] Kario K, Chen CH, Park S, Park CG, Hoshide S, Cheng HM (2018). Consensus document on improving hypertension management in Asian patients, taking into account Asian characteristics. Hypertension.

[36] Kario K, Shin J, Chen CH, Buranakitjaroen P, Chia YC, Divinagracia R (2019). Expert panel consensus recommendations for ambulatory blood pressure monitoring in Asia: the HOPE asia network. J Clin Hypertens (Greenwich).

[37] Kario K (2018). Nocturnal hypertension: new technology and evidence. Hypertension.

[38] Courand PY, Harbaoui B, Bècle C, Mouly-Bertin C, Lantelme P (2017). Plasma NT-proBNP mirrors the deleterious cardiovascular and renal continuum in hypertension. Eur J Prev Cardiol.

[39] Komori T, Eguchi K, Saito T, Hoshide S, Kario K (2017). Riser pattern is a novel predictor of adverse events in heart failure patients with preserved ejection fraction. Circ J.

[40] Kai H, Kudo H, Takayama N, Yasuoka S, Aoki Y, Imaizumi T (2014). Molecular mechanism of aggravation of hypertensive organ damages by short-term blood pressure variability. Curr Hypertens Rev.

[41] Kario K, Hoshide S, Mizuno H, Kabutoya T, Nishizawa M, Yoshida T (2020). Nighttime blood pressure phenotype and cardiovascular prognosis: practitioner-based nationwide JAMP study. Circulation.

